# Single-cell transcriptomics reveals mechanisms of *Galt* gene editing–induced liver injury involving HGF–VEGF–mediated intercellular signaling in mice

**DOI:** 10.3389/fcell.2025.1729321

**Published:** 2026-01-15

**Authors:** Zhihao Li, Ning Wang, Haolong Ruan, Qi Li, Xingling Zhang, Nian Liu, Yong Li, Lantao Gu, Pengpeng Yue, Honghao Yu

**Affiliations:** 1 Engineering Research Center of Rare Disease Prevention and Control, University of Guangxi, Guilin Medical University, Guilin, China; 2 Key Laboratory of Medical Biotechnology and Translational Medicine, Education Department of Guangxi Zhuang Autonomous Region, Guilin Medical University, Guilin, China; 3 Guangxi Key Laboratory of Drug Discovery and Optimization, Guilin Medical University, Guilin, China; 4 College of Intelligent Medicine and Biotechnology, Guilin Medical University, Guilin, China; 5 Guilin Hospital of Traditional Chinese Medicine, Guangxi University of Chinese Medicine, Nanning, China

**Keywords:** CRISPR/Cas9 system, galactosemia, *Galt* gene editing, liver injury, single-cell transcriptomics

## Abstract

Galactosemia, a genetic disorder caused by mutations in the human *GALT* gene, often leads to multi-organ damage, with liver injury being particularly prominent. To elucidate the molecular mechanisms of *Galt* in liver injury, this study employed the CRISPR/Cas9 system to construct a *Galt* (c.847 + 1G > T) gene-edited mouse (GAL mouse) model. Quantitative Real-time PCR and Western blotting revealed a significant reduction of *Galt* gene in GAL mice. Elevated liver index, serum ALT and AST levels, and H&E staining results indicated significant hepatocyte edema in GAL mice, suggesting a pronounced liver injury phenotype. Single-cell transcriptomics further unveiled significant changes in hepatocyte subtype proportions, with downregulation of metabolism-related genes and upregulation of immune-related genes. Cell communication analysis demonstrated that the communication of HGF and VEGF signaling pathways was significantly enhanced following *Galt* gene editing. The enhancement of HGF and VEGF signaling pathways may lead to hepatocyte edema, thereby causing liver injury. The GAL mouse model constructed in this study not only revealed the crucial roles of the *Galt* gene in liver metabolism, immune regulation, and cell communication, but also provided new insights into the pathogenesis of galactosemia and potential therapeutic targets.

## 1 Introduction

Galactosemia is a rare inherited metabolic disorder primarily caused by mutations in the *GALT* gene (galactose-1-phosphate uridylyltransferase) (Succoio et al., 2022; Tang et al., 2014). The disease is classified into three subtypes, with Galactosemia type I (GAL I) being the most common and severe (Demirbas et al., 2018). Galactose, an important carbohydrate, serves as a crucial energy source in neonates and plays a vital role in early development (Badiu Tişa et al., 2022). However, in individuals with GAL I, mutations in the *GALT* gene lead to the abnormal accumulation of galactose-1-phosphate (Wang et al., 2024), triggering acute multi-organ damage, particularly affecting the liver, kidneys, and nervous system (Lak et al., 2020). Without early intervention, these individuals may experience severe complications, including neonatal death.

Although the incidence of acute complications has significantly decreased with neonatal screening and early dietary management, many patients still face a range of long-term chronic issues as they grow, such as cognitive impairments, speech delays, and reproductive system dysfunction. This highlights the fact that current therapeutic approaches are insufficient to fully reverse the complex pathological processes caused by *GALT* gene mutations. Notably, the liver, as a central organ in galactose metabolism, undergoes structural and functional changes in galactosemia, but these alterations have not been thoroughly elucidated in existing studies.

Currently, various animal models of galactosemia have been established using mice (Chen et al., 2017), *Drosophila* (Kushner et al., 2010), and zebrafish (Haskovic et al., 2020). However, these models have limitations in accurately mimicking the complex pathology of the human disease, particularly in terms of reflecting liver-specific damage caused by *GALT* gene mutations. Therefore, it is crucial to develop models that more closely resemble human pathology. Consequently, we utilized CRISPR/Cas9 gene editing technology to precisely modify the *Galt* gene in mice, constructing an animal model that better simulates human galactosemia (Li et al., 2025). Unlike traditional gene editing models, CRISPR/Cas9 allows for more precise mutation design at the genetic level, thus recapitulating key pathological features of human galactosemia.

In this study, we applied single-cell transcriptomics (scRNA-seq) to conduct an in-depth analysis of the cellular composition and functional status of the *Galt* gene-edited mice liver. Single-cell RNA sequencing enables the exploration of gene expression patterns across different cell types within complex tissues, helping to reveal the specific effects of galactosemia on liver tissue and uncovering the mechanisms of multi-organ damage triggered by *GALT* gene mutations. We aim to provide new insights into how *GALT* gene mutations affect liver injury at the cellular level and potential therapeutic targets.

## 2 Results

### 2.1 *Galt* gene editing (GAL) mouse model construction and phenotypic analysis

**FIGURE 1:**
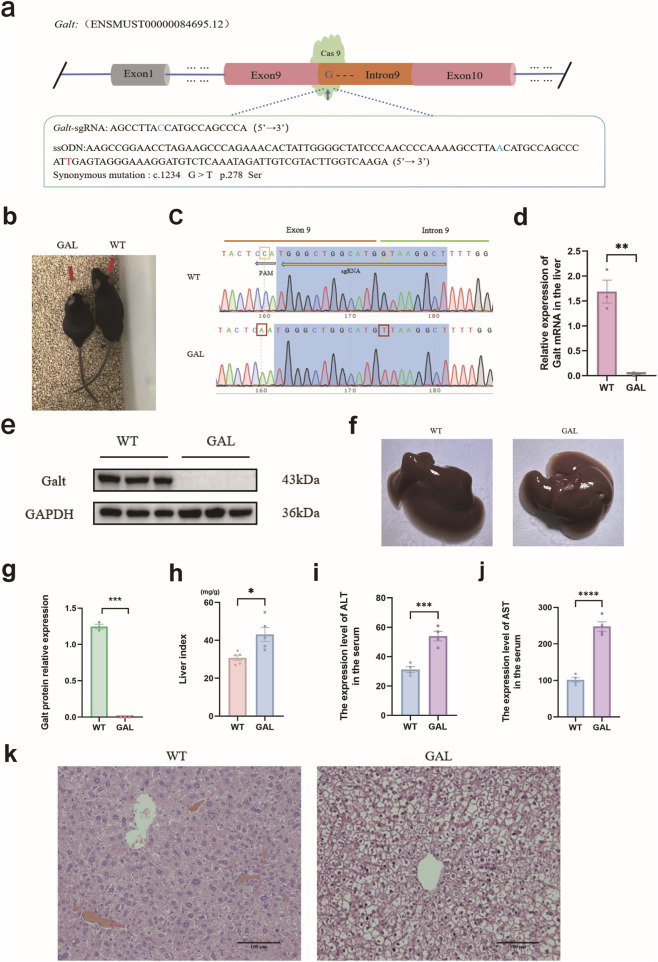
Analysis of *Galt* c.847 + 1G > T mutation and liver injury phenotypes in GAL mouse model. **(a)** Gene editing diagram of *Galt* c.847+1G > T. The Galt-sgRNA sequence is the complementary sequence of the target site. The blue letter C represents the base that complements *Galt* c.847+1G. The ssODN sequence is the complementary sequence of the target site, and the blue letter A represents the complementary sequence of the desired mutated base T. The red letter T represents a synonymous mutation base for *Galt* c.1234 G^10^. **(b)** Mouse obtained through breeding. (Left side is GAL mouse, right side is WT mouse). **(c)** Sanger sequencing diagram. The yellow box represents the base that has undergone pseudo-mutation, whereas the red box symbolize the bases that have been successfully mutated. **(d)** *Galt* mRNA relative expression level. **(e)** GALT protein expression detected by Western blotting. **(f)** Photographs of the livers from WT and GAL mice. **(g)** The statistic analysis of GALT protein expression. **(h)** Data analysis graph of liver index in WT and GAL mice. **(i,j)** Expression levels of ALT and AST in serum. **(k)** H&E staining of the liver pathology sections from both WT and GAL mice.

All experiments presented in this paper were conducted using the *Galt* (c. 847 + 1G>T) gene-edited mouse model, which was developed from prior laboratory work (Li et al., 2025). In brief, following procedures such as gene editing design, embryo injection and transfer, we successfully generated transplanted embryo mice (Figures 1a,b). DNA Sanger sequencing of the targeted region confirmed that the *Galt* c. 847 + 1G in transplanted embryo mice was successfully mutated to T (Figure 1c). After mating and breeding, we obtained the GAL mice (Figure 1b). No abnormal behaviors were observed in WT or GAL mice during routine monitoring throughout the experimental period. When compared to the wild-type (WT) mice, the GAL mice on were noticeably smaller, a characteristic consistent with the pathological features of galactosemia (Rokaitė et al., 2020). Compared to the WT mice, the expression levels of *Galt* mRNA and protein in the liver tissues of GAL mice were significantly diminished (Figures 1d,e,g). Observations of liver morphology from both groups indicated that the WT mice liver appeared firmer with a smoother surface, indicative of a healthy condition, whereas the GAL mice liver appeared flaccid, with a rough and irregular surface, lighter coloration, and a markedly larger volume. Further analysis of the liver index in both groups of mice demonstrated a significant increase in the liver index of GAL mice (Figures 1f,h). Additionally, the levels of ALT and AST in the serum of GAL mice were significantly higher than those in WT mice (Figures 1i,j), suggesting severe liver damage. Hematoxylin-Eosin (HE) staining corroborated these findings, showing hepatocyte swelling in the liver sections of GAL mice (Figure 1k). Collectively, these results indicate that the *Galt* c.847 + 1G > T mutation leads to substantial liver damage in GAL mice.

### 2.2 Remodeling of liver cell distribution and gene expression profile post-*Galt* gene editing

**FIGURE 2:**
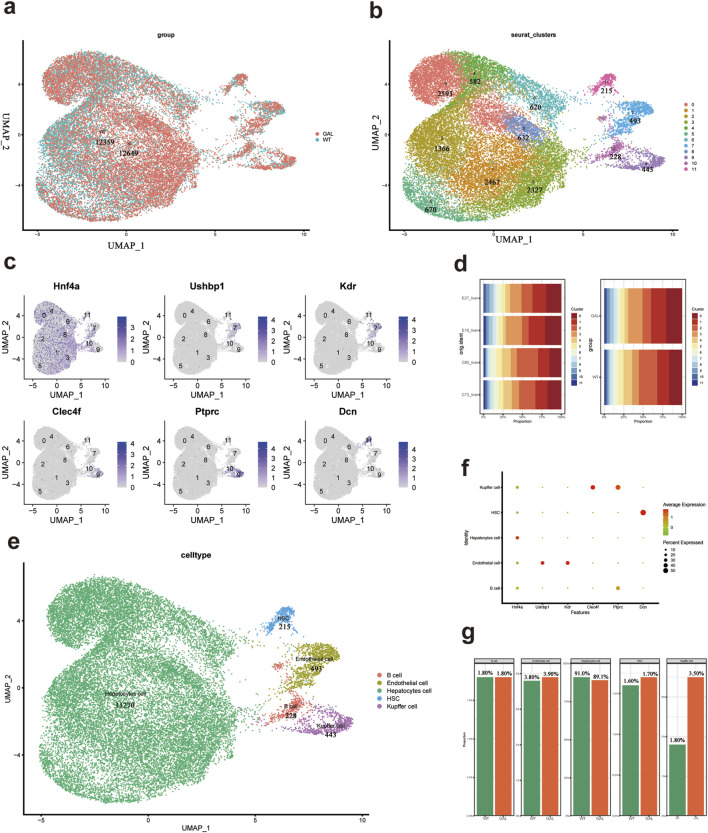
Cell types and their characteristic gene features in mice liver tissue. **(a)** UMAP scatter plot showing the uniform manifold approximation and projection of all cell types after annotation. **(b)** UMAP scatter plot of all 11 cell clusters after reclustering. **(c)** UMAP plots showing the expression of characteristic genes for each cell cluster. **(d)** Bar chart showing the proportion of each cell type in the GAL and WT groups (n = 2).**(e)** UMAP scatter plot of five annotated cell types. **(f)** Dot plot showing the expression of marker genes used for characterizing different cell types. **(g)** Bar chart showing the proportion of each cell type in the liver tissue of WT and GAL mice.

We integrated single-cell transcriptomic data from all samples and performed subsequent clustering analysis after quality control and batch effect removal. Using UMAP (Uniform Manifold Approximation and Projection) plots, we visualized the overall cell layout for both GAL and WT groups (Liang et al., 2022). Cells from the same sample were marked with the same color (Figure 2a). UMAP clustering of all cells divided them into 11 major clusters to identify different cell types (Figure 2b).

To better understand the composition of each cell cluster, we analyzed the expression of characteristic genes (Figure 2c). Representative genes such as Hnf4a, Ushbp1, Kdr, Clec4f, Ptprc, and Dcn were mapped in the UMAP plots to show their expression distribution. We then compared the distribution of cell clusters between the GAL and WT groups in different experimental samples (E27, E19, C53, and C72) (Figure 2d). Each colored block represents a specific cell cluster, with different colors corresponding to clusters numbered from 0 to 11. We observed that the major cell cluster distribution trends were similar between the experimental replicates of both groups, but there were differences in the distribution ratios of certain clusters, indicating potential effects of *Galt* gene editing on specific cell types.

To determine the identity of each cell cluster, we manually annotated the clusters based on their specific marker genes and biological functions. The classical marker gene Hnf4a was highly expressed in hepatocytes, Kdr was predominantly expressed in endothelial cells, and Clec4f was a hallmark gene for Kupffer cells. UMAP visualization was performed to project the cell clusters (Figure 2e), and these were categorized into five types of cells. The expression of characteristic markers in each cell type was visualized (Figure 2f). Specifically, typical hepatocyte markers, including Hnf4a, were highly expressed in eight clusters (0, 1, 2, 3, 4, 5, 6, 8). The expression of Kdr in cluster seven indicated endothelial cells, while Clec4f, a marker for Kupffer cells, was highly expressed in cluster 9. The stellate cell marker Dcn was predominantly expressed in cluster 11. Based on the expression distribution of these marker genes, we were able to more accurately identify the cell types within each cluster.

When comparing the proportion of different cell types in the liver tissue before and after *Galt* gene editing (Figure 2g), we found a significant increase in the proportion of Kupffer cells in the GAL mouse liver tissue, while the proportion of hepatocytes was slightly decreased. This suggests that *Galt* gene mutation may lead to the aggregation or proliferation of inflammatory cells, thus altering the composition of hepatocyte populations. Additionally, the proportions of hepatic stellate cells and endothelial cells were also increased in the GAL mice, suggesting a close correlation with immune responses and inflammatory states in the liver tissue.

### 2.3 Impact of *Galt* gene editing on cell types, gene expression, and cell communication

**FIGURE 3:**
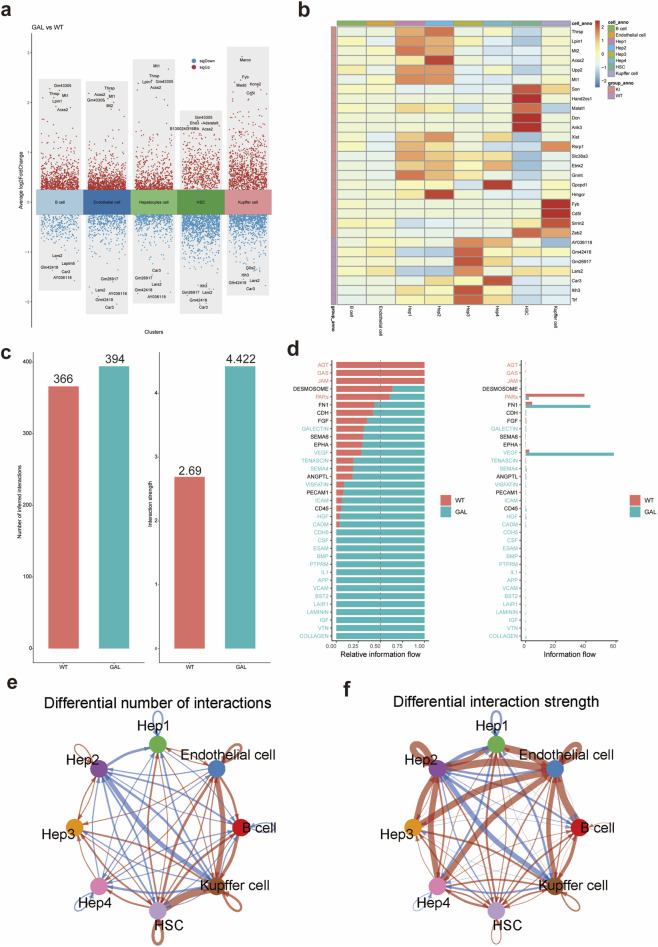
Differential gene expression analysis induced by *Galt* gene editing in mice liver tissue. **(a)** Volcano plot of differential gene expression showing the distribution of upregulated and downregulated genes between the GAL and WT groups.**(b)** Heatmap of differential gene expression, displaying differences in gene expression among hepatocytes, Kupffer cells, and endothelial cells in the GAL and WT groups. **(c)** Bar chart of the number of cell-cell interactions in WT and GAL groups. **(d)** Bar plot of enriched signaling pathways significantly elevated in the GAL group. **(e)** Network diagram illustrating differences in ligand–receptor pair numbers between cell clusters in the WT and GAL groups. **(f)** Network diagram depicting differences in intercellular communication strength between cell clusters in the WT and GAL groups.

We further investigated the effects of *Galt* gene editing on liver cell types, gene expression, and cell communication patterns, revealing significant changes across multiple aspects. First, volcano plots (Figure 3a) illustrate the differential gene expression between the GAL and WT groups, with upregulated genes marked in red and downregulated genes in blue. The volcano plot clearly shows that in GAL mice, the expression of hepatocyte metabolism-related genes significantly decreased, while the expression of inflammation-related genes in Kupffer cells and endothelial cells markedly increased. These changes suggest that *Galt* gene editing may lead to functional alterations in the liver. We further explored gene expression patterns across different cell types using a heatmap of differential genes (Figure 3b). The results revealed that immune-related genes were highly expressed in Kupffer cells and endothelial cells, while metabolism-related genes were expressed at lower levels in hepatocytes, further supporting the functional impact of *Galt* gene editing on gene expression in different cell types.

To investigate the impact of *Galt* gene editing on cell communication, we used the CellChat tool to analyze intercellular interactions between different cell types in the WT and GAL samples. In the analysis of the quantity and intensity of intercellular interactions between different cell populations (Figure 3c), we observed significant differences. The inferred number of intercellular interactions in the WT group was 366, while in the GAL group it was 394, indicating that the GAL group had a notably higher number of interactions compared to the WT group. Furthermore, the strength of these interactions was also significantly greater in the GAL group, suggesting that gene editing triggered more cell communication activity, with both the quantity and intensity of interactions being more pronounced in the GAL group.

Subsequent enrichment pathway analysis (Figure 3d) revealed significant differences in communication pathways between the WT and GAL groups. Specifically, when the ratio of the summed pathway probabilities between the GAL and WT groups was less than 0.95 with a p-value <0.05, the communication intensity of that pathway was significantly higher in the WT group. Conversely, when the ratio of the summed pathway probabilities between the GAL and WT groups was greater than 1.05 with a p-value <0.05, the communication intensity of that pathway was significantly higher in the GAL group. Notably, in the VEGF (vascular endothelial growth factor) and HGF (hepatocyte growth factor) signaling pathways, the information flow in the GAL group was significantly enhanced (Zhao et al., 2022). These pathways are likely activated after gene editing and may be involved in critical processes such as liver repair, angiogenesis, and cell migration.

Moreover, to identify interactions between cell populations showing significant changes, we compared the number and intensity of interactions between different cell populations (Figures 3e,f). The number and intensity of cell interactions were significantly higher in the GAL group than in the WT group, particularly in the communication between immune cells and hepatocytes. These results indicate that *Galt* gene editing not only alters the composition and function of cell types but also influences overall liver function by enhancing interactions between specific cell populations.

### 2.4 Impact of *Galt* gene editing on hepatocyte subtype distribution and function

**FIGURE 4:**
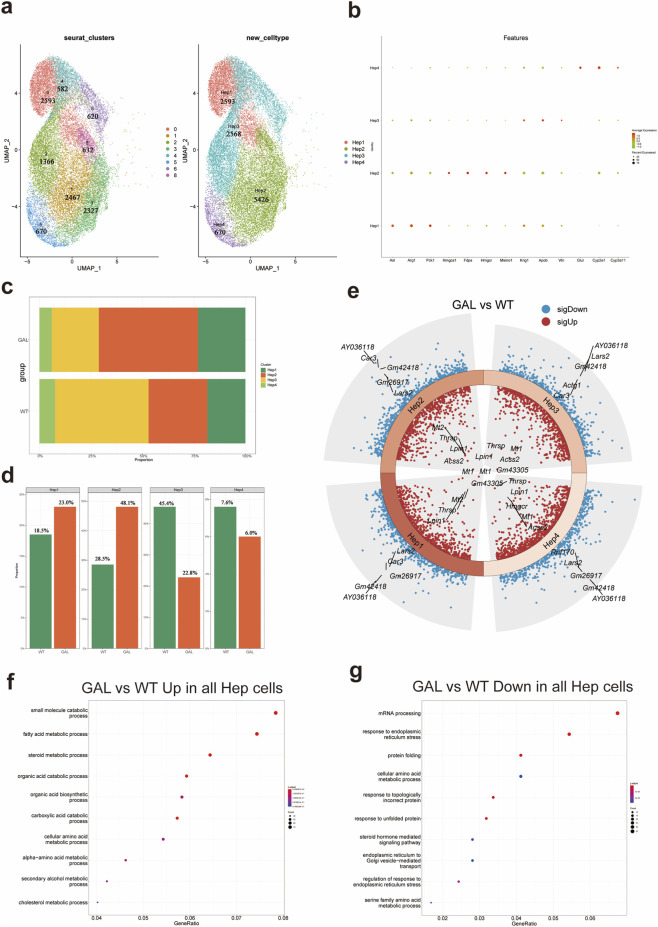
Effect of *Galt* gene editing on hepatocyte subtypes in mice liver tissue. **(a)** UMAP plot showing four hepatocyte subtypes (Hep1 to Hep4), categorized based on characteristic marker genes. **(b)** Dot plot of marker gene expression in different hepatocyte subtypes in GAL and WT groups. **(c)** Bar chart of the cell count of each hepatocyte subtype (Hep1 to Hep4) in the GAL and WT groups. **(d)** Bar chart of the relative proportions of each hepatocyte subtype in the GAL and WT groups. **(e)** Heatmap of differentially expressed genes, showing gene expression differences between GAL and WT groups in Hep1 and Hep3 subtypes. **(f)** GO enrichment analysis indicating upregulated inflammation-related pathways in overall hepatocytes in the GAL group. **(g)** GO enrichment analysis showing downregulated metabolism-related pathways in overall hepatocytes in the GAL group.

To further analyze the impact of *Galt* gene editing on liver cells, we classified hepatocytes into four main subtypes: Hep1, Hep2, Hep3, and Hep4 (Figure 4a), based on single-cell transcriptomic data and key marker genes. Specifically, Hep1 consists of cluster 0. Hep2 is merged from clusters 1, 3, and 8. Hep3 is merged from clusters 2, 4, and 6. Hep4 consists of cluster 5. Each subtype was categorized according to its unique gene expression profile. The characteristic genes of the Hep1 subtype were metabolic-related genes such as Asl, Arg1, and Pck1. The Hep2 subtype exhibited higher expression of cholesterol synthesis-related genes, including Hmgcs1, Fdps, Hmgcr, and Msmo1. The Hep3 subtype showed immune-regulatory characteristics, with higher expression of Kng1, Apob, and Vtn. The Hep4 subtype displayed elevated expression of detoxification and oxidative metabolism-related genes such as Glul, Cyp2e1, and Cyp3a11. A dot plot (Figure 4b) visualized the gene expression patterns of these characteristic genes, further illustrating the expression of typical markers in each subtype. Using this classification, we investigated the dynamic changes of hepatocyte subtypes under the background of *Galt* gene editing.

When comparing the hepatocyte subtype composition between the GAL and control WT groups, quantitative analysis (Figures 4c,d) revealed that in the GAL group, the proportions of Hep1 and Hep2 subtypes were significantly increased. The proportion of Hep1 cells in the WT group was 18.5%, but increased to 23.0% in the GAL group, suggesting that metabolism-related hepatocytes exhibit enhanced adaptive capacity under *Galt* gene editing conditions. The proportion of Hep2 cells rose from 28.5% in the WT group to 48.1% in the GAL group, possibly reflecting changes in metabolic and immune regulatory functions. In contrast, the proportions of Hep3 and Hep4 subtypes were significantly reduced. The proportion of Hep3 cells decreased from 45.4% in the WT group to 22.8% in the GAL group, suggesting that the substantial reduction in Hep3 cells may be related to altered immune regulatory cell functions due to *Galt* gene editing.

To further understand the molecular mechanisms behind the changes in hepatocyte subtype numbers, we performed differential gene expression analysis for each subtype (Figure 4e). The results showed that in both Hep1 and Hep2 subtypes, several metabolism-related genes were significantly downregulated, indicating that these subtypes may experience inhibited metabolic functions when responding to metabolic stress. However, oxidative stress-related genes were significantly upregulated in these subtypes, suggesting that *Galt* gene editing may induce an enhanced adaptive response in these cells to counteract metabolic imbalance. In contrast, the Hep3 and Hep4 subtypes exhibited higher expression of genes related to small molecule metabolism and lipid metabolism, suggesting that these cells adapt to the stress caused by the loss of Galt by enhancing specific metabolic pathways.

Based on these differential gene expression changes, we further conducted GO (Gene Ontology) functional enrichment analysis to explore the biological processes and signaling pathways potentially involved (Figures 4f,g). The results indicated that the upregulated pathways in hepatocytes were significantly enriched in key metabolic processes such as cholesterol metabolism and fatty acid metabolism, suggesting that in the absence of Galt, the liver may adapt to metabolic stress by activating these metabolic pathways. This change suggests a compensatory response of the liver to metabolic challenges, although the role of these pathways in overall metabolic regulation requires further investigation. In contrast, the downregulated pathways in hepatocytes were primarily associated with endoplasmic reticulum stress response and protein folding, with significantly reduced activity in these pathways in the GAL group. The downregulation of these metabolic pathways may impair the liver’s ability to respond to metabolic stress and maintain protein quality control, thereby exacerbating the effects of *Galt* gene editing on liver function.

These results indicate that *Galt* gene editing not only significantly affects the distribution of hepatocyte subtypes but also influences the specific biological functions of each subtype through the regulation of key gene expression. These findings highlight the critical role of the *Galt* gene in maintaining hepatocyte homeostasis and function.

### 2.5 Impact of *Galt* gene editing on cell communication networks of Hep3 and Hep4 subtypes

We systematically investigated the impact of *Galt* gene editing on the intercellular communication networks among different cell types in the liver of mice at the single-cell level, with a particular focus on the changes in the Hepatocyte Growth Factor (HGF) and Vascular Endothelial Growth Factor (VEGF) signaling pathways. In the Hep3 and Hep4 hepatocyte subtypes, significant differences in intercellular communication intensity and patterns were observed between the GAL and WT groups.

**FIGURE 5:**
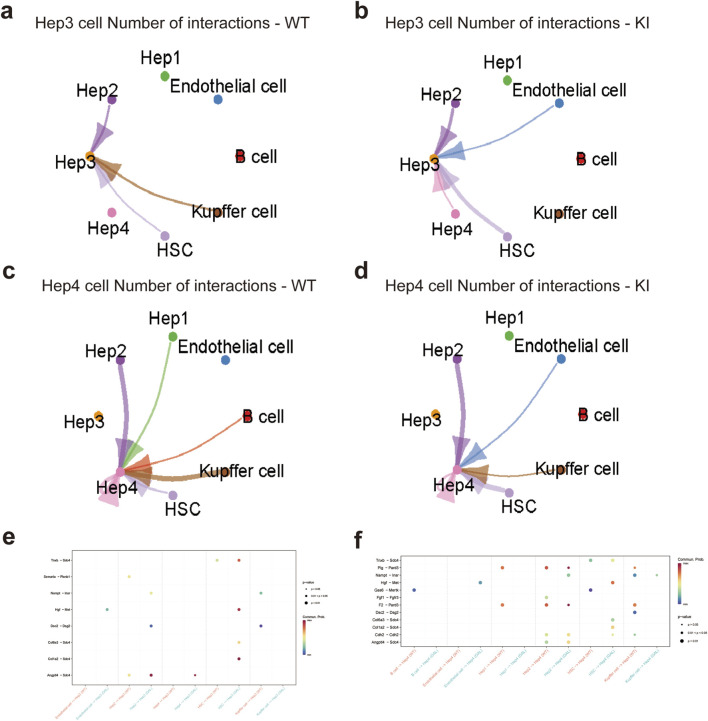
Effects of *Galt* gene editing on cell communication networks of Hep3 and Hep4 subtypes in mice liver tissue. **(a)** Ligand-receptor interaction network for Hep3 cells in the WT group. **(b)** Ligand-receptor interaction network for Hep3 cells in the GAL group. **(c)** Ligand-receptor interaction network for Hep4 cells in the WT group. **(d)** Ligand-receptor interaction network for Hep4 cells in the GAL group. **(e)** Dot plot of ligand-receptor pairs between Hep3 subtype and different cell types, indicating communication patterns in specific signaling pathways. **(f)** Dot plot of ligand-receptor pairs between Hep4 subtype and different cell types, indicating communication patterns in specific signaling pathways.

Firstly, intercellular interactions in the Hep3 and Hep4 subtypes were generally enhanced in the GAL group (Figures 5a–d). Compared to the WT group, Hep3 subtype interactions with other cell types, such as endothelial cells and hepatic stellate cells (HSCs), were significantly increased. Similar trends of enhanced communication were also observed for Hep4 subtypes. Ligand-receptor pair analysis further revealed significant alterations in several key signaling pathways in the GAL group (Figures 5e,f). Specifically, the HGF and VEGF signaling pathways showed stronger activity in communication between the Hep3 and Hep4 subtypes and other cell types, particularly in the enhanced interactions between endothelial cells, HSCs, and hepatocytes.

### 2.6 Impact of *Galt* gene editing on HGF and VEGF signaling pathways

**FIGURE 6:**
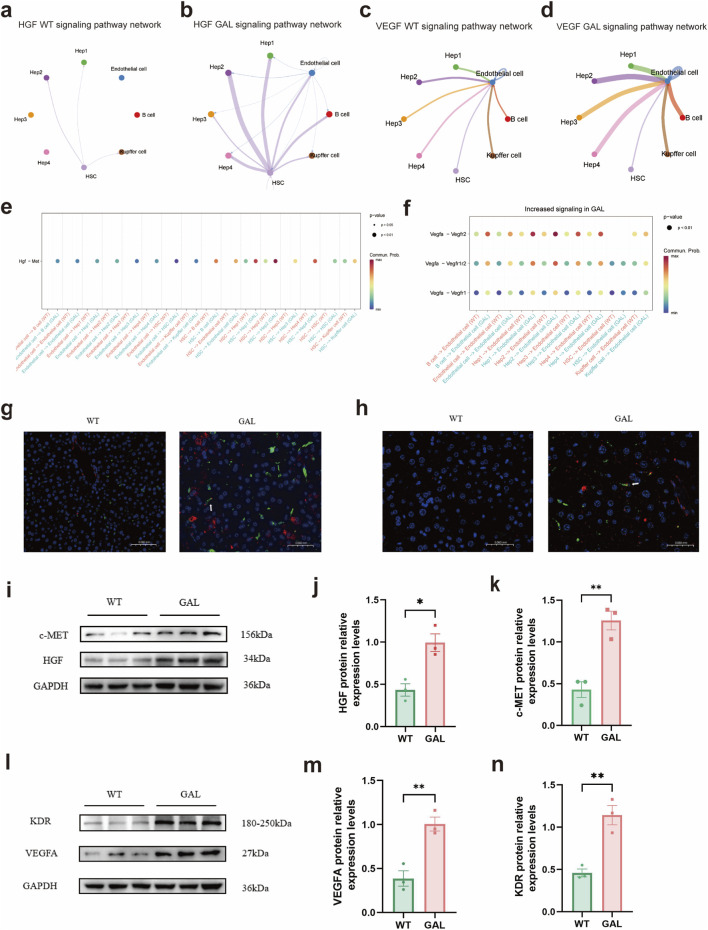
Effects of *Galt* gene editing on HGF and VEGF signaling pathways in mice liver tissue. **(a)** Cell-cell communication network of the HGF pathway in the GAL group. **(b)** Cell-cell communication network of the HGF pathway in the WT group. **(c)** Cell-cell communication network of the VEGF pathway in the GAL group. **(d)** Cell-cell communication network of the VEGF pathway in the WT group. **(e)** Dot plot of differential expression in cell-cell communication in the HGF pathway between GAL and WT groups. **(f)** Dot plot of differential expression in cell-cell communication in the VEGF pathway between GAL and WT groups. **(g)** Representative immunofluorescence (IF) images of liver tissues from WT and GAL mice showing the expression of HGF (red) and c-MET (green), with nuclei counterstained by DAPI (blue). **(h)** Representative immunofluorescence (IF) images of liver tissues from WT and GAL mice showing the expression of VEGFA (red) and KDR (green), with nuclei counterstained by DAPI (blue). **(i)** WB analysis of HGF and c-MET protein expression in WT and GAL liver tissues. **(j,k)** Statistical analysis of HGF and c-MET protein expression levels based on WB results. (For interpretation of the references to color in this figure legend, the reader is referred to the Web version of this article.) **(l)** WB analysis of VEGFA and KDR protein expression in WT and GAL liver tissues. **(m,n)** Statistical analysis of VEGFA and KDR protein expression levels based on WB results.

After investigating the extensive effects of *Galt* gene editing on the communication network of hepatocyte subtypes (Figure 5), we further explored how these changes impacted specific signaling pathways, particularly the HGF and VEGF pathways. Our analysis revealed a significant increase in the communication of the HGF-c-Met ligand-receptor pair among different cell types in the GAL gene-edited group (Figures 6a, b#). Notably, the communication strength of the HGF pathway between Hep3 and Hep4 subtypes and endothelial cells, as well as hepatic stellate cells (HSCs), was markedly enhanced. This finding was supported by the differential expression dot plot of the HGF pathway, which showed a statistically significant increase in HGF signaling among multiple cell types in the GAL group (Figure 6e). Overactivation of the HGF signaling pathway can lead to abnormal deposition of the extracellular matrix (ECM), thereby affecting the normal function of hepatocytes and causing dysregulation of inflammatory responses and hepatocyte edema (De Silva et al., 2017; Xia et al., 2025).

The VEGF signaling pathway exhibited a similar trend. Compared with the WT group, the communication of the VEGF signaling pathway between Hep3 and Hep4 subtypes and endothelial cells, as well as HSCs, was significantly enhanced in the GAL group (Figures 6c,d). The differential expression dot plot of the VEGF signaling pathway further confirmed this, showing a significant increase in the communication of VEGF ligand-receptor pairs among these cell types in the GAL gene-edited group (Figure 6f), especially in communication with endothelial cells. Upon activation via its receptor (e.g., VEGFA), VEGF primarily promotes the proliferation and migration of vascular endothelial cells, improving liver microcirculation. However, overactivation of VEGF can lead to abnormal ECM deposition, affecting the normal function of hepatocytes and potentially increasing vascular permeability, thereby promoting the infiltration of inflammatory cells (Lee et al., 2025; Tolstanova et al., 2010). Moreover, the intercellular communication strength of the VEGF pathway was significantly increased in the GAL group.

The bar charts of communication strength for the HGF and VEGF pathways clearly demonstrated the differences between the GAL and WT groups (Figures 6g,h). In both the HGF and VEGF pathways, the communication strength between Hep3 and Hep4 subtypes and endothelial cells, as well as HSCs, was significantly enhanced in the GAL group. These results further supported the notion that *Galt* gene editing can enhance the expression of the HGF and VEGF signaling pathways.

Immunofluorescence and Western blot experiments also confirmed the overexpression of the HGF and VEGFA signaling pathways in the livers of GAL mice (Figures 6i–n). These findings suggest that *Galt* gene editing may cause liver injury in mice by enhancing the HGF and VEGF signaling pathways.

In conclusion, our study demonstrated that *Galt* gene editing significantly enhanced the communication of the HGF and VEGF signaling pathways among different cell types in the liver, particularly between endothelial cells, HSCs, and hepatocyte subtypes (Hep3 and Hep4). These findings highlight the critical role of the *Galt* gene in regulating the normal structure and function of the liver and provide new insights for potential targeted interventions in the treatment of liver diseases.

### 2.7 Effects of *Galt* gene editing on functional pathways in Kupffer cells and hepatic stellate cells

To further investigate the impact of *Galt* gene editing on non-parenchymal liver cell functions, we performed GO enrichment analysis on Kupffer cells and hepatic stellate cells, showing significant changes in both upregulated and downregulated pathways.

**FIGURE 7:**
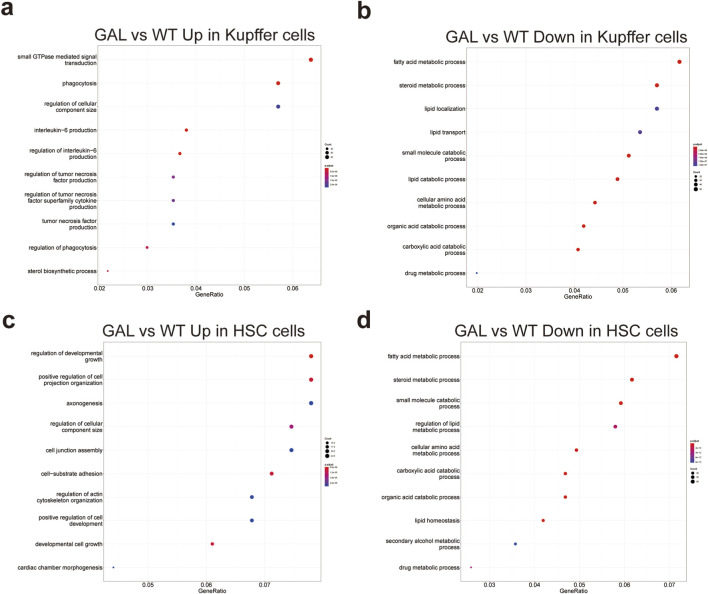
Effects of *Galt* gene editing on functional pathways in Kupffer cells and hepatic stellate cells. **(a)** GO enrichment analysis indicating upregulated inflammation-related pathways in Kupffer cells in the GAL group. **(b)** GO enrichment analysis indicating downregulated metabolism-related pathways in Kupffer cells in the GAL group. **(c)** GO enrichment analysis showing upregulated inflammation-related pathways in hepatic stellate cells in the GAL group. **(d)** GO enrichment analysis showing downregulated metabolism-related pathways in hepatic stellate cells in the GAL group.

In Kupffer cells, compared to the WT group, the GAL group exhibited upregulation in pathways such as small GTPase-mediated signal transduction, phagocytosis, regulation of cellular component size, and interleukin-6 (IL-6) signaling (Figure 7a). The increased activity in these pathways suggests an enhancement of immune regulation and phagocytic functions in response to inflammation and metabolic imbalance triggered by Galt deficiency. Conversely, downregulated pathways in Kupffer cells were primarily enriched in fatty acid metabolism, steroid metabolism, small molecule metabolism, lipid transport, and drug metabolism (Figure 7b), indicating a reduction in metabolic activity, particularly in lipid metabolism, under *Galt* gene deletion.

In hepatic stellate cells, the upregulated pathways in the GAL group included those associated with the regulation of developmental growth, cell junction assembly, cell-matrix adhesion, and actin cytoskeleton organization (Figure 7c). These increases might reflect an enhancement of cellular structure and developmental functions, implying a potential role of hepatic stellate cells in liver repair processes following *Galt* deletion. Conversely, downregulated pathways were predominantly related to steroid metabolism, small molecule metabolism, lipid homeostasis, and drug metabolism (Figure 7d), further indicating a reduction in metabolic activity within hepatic stellate cells.

In summary, *Galt* gene editing led to significant pathway remodeling in Kupffer cells and hepatic stellate cells, manifesting as enhanced immune regulation, cellular structure maintenance, and altered metabolic functions. This highlights the critical role of the *Galt* gene in regulating non-parenchymal cell responses in the liver.

## 3 Discussion

This study delves into the crucial role of the *Galt* gene in maintaining liver health. By constructing the GAL mouse model and employing a suite of techniques including HE staining, immunofluorescence, Western blotting, and single-cell transcriptome analysis, we have unveiled the severe damage that *Galt* gene editing inflicts upon liver structure and function. It reshapes the cellular landscape, disrupts communication networks between cells, and alters critical signaling pathways.

### 3.1 Pathological and cellular changes in the GAL mouse model

When the *Galt* gene is edited in the liver, it sets off a powerful inflammatory response—a clear sign of liver injury. Elevated liver index, soaring levels of serum ALT and AST, and H&E staining all point to extensive swelling of hepatocytes in the GAL mice liver. Mice in the GAL group exhibited significant liver damage and growth restriction, with pathological features similar to those observed in the *Galt* gene-edited mouse model studied by Manshu Tang et al. (Tang et al., 2014). Single-cell transcriptome analysis further reveals shifts in the makeup of major liver cell populations. Hepatocytes take a slight dip, while Kupffer cells, endothelial cells, and hepatic stellate cells surge. These changes underscore *Galt* vital role in keeping liver tissue balanced and hint at its influence on the liver immune environment.

### 3.2 Impact on hepatocyte subtypes and metabolic pathways

Our subtype analysis shows that *Galt* gene editing dramatically affects specific hepatocyte subtypes. The metabolically active Hep1 and Hep2 subtypes increase, while the immune-regulating Hep3 subtype dwindles. Differential expression analysis uncovers downregulation of key metabolic genes like Asl, Arg1, and Pck1 in hepatocytes, while immune-related genes in Kupffer and endothelial cells spike. This suggests that *Galt* deficiency might hamper metabolic functions while ramping up immune responses. Gene Ontology (GO) enrichment analysis highlights significant changes in metabolic and stress response pathways in *Galt* editing cells. Fatty acid and cholesterol metabolism pathways in hepatocytes ramp up, likely a compensatory response to metabolic stress. Meanwhile, downregulation of endoplasmic reticulum (ER) stress responses and protein folding pathways hints at potential issues in handling metabolic stress and maintaining protein quality control.

### 3.3 Intercellular communication and signaling pathways

CellChat analysis shows a marked increase in intercellular communication, especially in the HGF and VEGF signaling pathways. The enhanced HGF-Met ligand-receptor pair communication, particularly between hepatocyte subtypes and endothelial or hepatic stellate cells, could lead to uncontrolled inflammatory responses and hepatocyte swelling. Similarly, the upregulation of VEGF signaling, which boosts vascular permeability and ECM deposition, might contribute to hepatocyte dysfunction and inflammation (Zhao et al., 2022; Hida et al., 1999). Additionally, the synergistic effects between the HGF and VEGF pathways may intensify these responses, thereby causing hepatocyte swelling and inflammatory reactions. This indicates that the *Galt* gene may maintain the normal structure and function of the liver by regulating the expression of the HGF and VEGF pathways.

The intensified intercellular signaling likely reflects a compensatory mechanism (Huang et al., 2022), with the liver attempting to mitigate the damage caused by *Galt* gene deficiency by enhancing critical signaling pathways. However, if this compensation is excessive or misdirected, it may exacerbate hepatic inflammation and injury. Consequently, an in-depth comprehension of HGF and VEGF pathways may offer crucial insights for devising novel therapeutic approaches, particularly in the modulation of liver inflammation and facilitation of hepatic regeneration.

### 3.4 Comparison with other *Galt* gene editing models

Compared to other *Galt* gene editing models, such as those in *Drosophila* (Daenzer et al., 2016) or zebrafish (Vanoevelen et al., 2018), our GAL mouse model offers a more detailed look at liver-specific pathology. While previous models have shown the general impact of *Galt* deficiency on development and metabolism, our study zooms in on the liver. We reveal specific changes in cellular composition, signaling pathways, and intercellular communication networks that are not fully captured in non-mammalian models. The significant changes in hepatocyte subtypes and the upregulation of HGF and VEGF pathways highlight the complexity of *Galt*’s role in maintaining liver homeostasis, which might be overlooked in simpler model organisms.

### 3.5 Conclusion and future directions

This study, through single-cell transcriptomics and cell communication analysis, reveals the significant impact of *Galt* gene editing on mice liver metabolism, immunity, and fibrosis. The absence of the *Galt* gene not only leads to the remodeling of liver cell composition and gene expression profiles but also profoundly affects intercellular communication networks. The notable upregulation of key signaling pathways such as VEGF and HGF may be crucial factors leading to hepatocyte edema and inflammatory cell infiltration, thereby exacerbating liver injury.

Future research can further explore how to alleviate liver injury caused by *Galt* gene editing by targeting these key signaling pathways. This study provides new insights into the role of the *Galt* gene in liver metabolism and immune regulation and offers potential targets for the treatment strategies of galactosemia.

### 3.6 Limitation

A limitation of this study is the low capture of cholangiocytes and lymphocytes, which failed to form clear clusters. Thus, our dataset does not fully capture the hepatic microenvironment. Consequently, cell - cell communication analysis was restricted to five major cell types, without assessing interactions with cholangiocytes or lymphocytes. Conclusions regarding immune regulation and intercellular signaling should therefore be interpreted with caution.

The lack of specialized methods for ambient RNA and doublet removal represents a technical limitation. Nevertheless, the high data quality and clear separation of cell populations support the robustness of our conclusions.

## 4 Materials and methods

### 4.1 Animals

All mice used in this study were C57BL/6J, with sexes randomly assigned, aged 8–10 weeks, and weighing 18–22 g. They were fed SPF-grade laboratory breeding chow and fasted for 12 h prior to euthanasia. All mice used in this study were SPF-grade and housed under standard SPF conditions. All experiments involving mice were approved by the Institutional Animal Care and Use Committee of Guilin Medical University (Permission code: GLMC202103279).

### 4.2 Design of sgRNAs and ssODN targeting *Galt*

The mouse *Galt* gene sequence (ENSMUST00000084695.12) was analyzed, and *Galt* c.847 + 1G was identified as the first base of intron 9, a conserved splice site. In this study, we aimed to introduce a mutation at *Galt* c.847 + 1G to T, which would disrupt normal exon splicing and result in a truncated protein. Based on previous studies on efficient ssODN-mediated targeting, we designed a single-guide RNA (sgRNA) and a single-stranded oligodeoxynucleotide (ssODN) targeting the *Galt* c.847 + 1G site (Figure 1a). The ssODN served as a homologous recombination template, with the c.847 + 1G mutation to T, alongside a secondary mutation at c.1234 G in exon 9 (G to T). This change altered the CRISPR/Cas9 PAM sequence, preventing Cas9 cleavage after successful mutation while leaving the codon for p.278 Ser unaffected (Figure 1c).

### 4.3 Construction and genotyping of GAL mouse models

The sgRNA and ssODN were chemically synthesized and dissolved in water for microinjection into fertilized eggs. Cas9 protein was purchased from Thermo Fisher (A50575). Superovulation of female mouse was performed to collect fertilized eggs, which were subsequently fertilized *in vitro*. The sgRNA (200 ng/μl), ssODN (100 ng/μl), and Cas9 protein (200 ng/μl) were microinjected into the cytoplasm of zygotes at the pronuclear stage. After injection, the embryos were cultured to the 2-cell stage before being implanted into the oviducts of pseudopregnant female mice. This procedure resulted in the establishment of *Galt* gene-edited mouse models.

For the analysis of *Galt* genotype in obtained mice, tail tissues collected from mice were digested in lysis buffer (0.4 M NaCl, 2 µM EDTA, 1% SDS, 10 µM Tris-HCl, and 100 μg/ml Proteinase K) overnight. Genomic DNA of the sample was extracted from the lysate with phenol-chloroform and recovered by ethanol precipitation. Genomic DNA was used as templates for PCR amplification of the targeted region with specific primers (Li et al., 2025). The primers used are listed in Supplementary Table S1. PCR products were purified using a cleanup kit (AP-PCR-50, Axygen, New York, USA). The purified PCR product used for Sanger sequencing. Homozygous GAL mouse model with *Galt* c.847 + 1G>T was obtained by genotypic analysis and breeding.

### 4.4 Analysis of *Galt* gene expression levels

In this study, mRNA and protein expression levels of *Galt* gene in liver tissues of GAL mice were detected by Quantitative RT-PCR (qPCR) and Western blotting (WB) (Li et al., 2025).

Total RNAs for qPCR analysis were extracted from liver tissue of GAL mice and wild type (WT) mice. The cDNA was synthesized based on 1 μg RNA template with an available RNA extraction kit (TaKaRa. Biotech. Co. Ltd., Dalian, China). qPCR was set up in 10 μl reaction mixtures which contain 2× SYBR (TaKaRa. Biotech. Co. Ltd., Dalian, China) 5 μl, extraction of cDNA 1 μl, forward primer 0.5 μl, reverse primer 0.5 μl, ddH_2_O 3 μl. Reaction procedure were as follows: 95 °C for 3 min and then 40 cycles 95 °C for 10 s, 55 °C for 30 s. All expression levels were normalized to GAPDH. The double DCt method was used as measurement of the expression level. The fluorescence signal was collected every 0.5 °C for 10 s. The primers used are listed in Supplementary Table S2.

The liver from GAL mice and WT mice were used to evaluate GALT protein level using Western blotting. In brief, the tissues were lysed by RIPA lysis buffer (Bestbio, China) with protease inhibitors at 4 °C. After lysis, we collected supernatants by centrifugation at 13,800×g for 15 min at 4 °C. Equal amounts of protein (50 μg) were separated by SDS-PAGE gel, along with protein weight marker using electrophoresis. Then the proteins were transferred to PVDF membranes and incubated with primary antibody against GALT (ab178406, Abcam, Cambridge,Englan; 1:2000) and GAPDH (GB11002, Wuhan Sevier Biotechnology Co., LTD, China; 1:5000) at 4 °C overnight. After incubation, the membranes were washed and reacted with anti-mouse or rabbit secondary antibodies (R&D, USA). The membranes were incubated with the ECL (Easysee Western blot Kit, China) and visualized with an Imaging System (Bio-Rad, Universal Hood II, USA).

### 4.5 Hematoxylin and eosin (H&E) staining

Liver tissues from GAL and WT mice were fixed in 4% formalin, paraffin-embedded, sectioned at 4 μm, deparaffinized, and stained with hematoxylin and eosin (HE) using a commercial kit (G1120, Beijing Solarbio Science & Technology Co., Ltd.). Sections were sequentially rehydrated through a descending ethanol series (100%, 95%, 85%, 75%; 3 min each), rinsed in distilled water for 2 min, and stained with hematoxylin for 4 min. After a distilled water rinse, sections were differentiated for 40 s and washed in distilled water for 8 min. Following eosin counterstaining for 1 min, sections were rapidly dehydrated through an ascending ethanol series (75%, 85%, 95%, 100%; 2–3 s each), cleared in xylene twice (1 min each), and mounted with neutral balsam. The slides were then observed under bright-field microscopy using an Olympus DP72 imaging system.

### 4.6 Single-cell preparation

Liver tissue samples were obtained from two GAL mice and two wild-type (WT) mice. The samples were first washed in pre-chilled RPMI 1640 medium to remove impurities and residual blood. Subsequently, tissue dissociation was performed using Seek-Gene Tissue Dissociation Reagent A (Catalog number: Seekone K01301-30). To further process the samples, red blood cells were removed using a red blood cell lysis reagent (R1010), followed by cell counting and viability assessment. Based on these evaluations, additional processing steps were applied, including the use of magnetic bead kits (Miltenyi 130-109-398/130-090-101) to eliminate cell debris and dead cells. During this process, the cell suspension was washed twice with RPMI 1640 medium, and the final cell suspension was resuspended in 1× PBS containing 0.04% bovine serum albumin (BSA), adjusting the cell density to 1 × 10^6^ cells per milliliter.

### 4.7 Single-cell RNA sequencing library construction and sequencing

Single-cell RNA sequencing libraries were constructed using the SeekOne® Digital Droplet Single Cell Library Construction Kit (SeekGene Catalog number K00202). Cells from each sample were first mixed with reverse transcription reagents and then loaded into the sample wells of the SeekOne® chip. Highly stable droplet emulsions were generated using unique barcode hydrogel beads (BHBs) and oil-phase technology. Reverse transcription was carried out at 42 °C for 90 min, and the reaction was terminated by heating at 80 °C for 15 min. The generated cDNA was separated and purified from the droplets and amplified by PCR. After amplification, the cDNA was fragmented, end-repaired, and A-tailed, followed by the ligation of sequencing adapters. The final library was amplified by index PCR and purified using SPRI beads. Library construction was completed, and sequencing was performed on the Illumina NovaSeq 6000 platform with paired-end 150 bp (PE150) sequencing.

### 4.8 Raw data processing

The raw single-cell sequencing data were processed using a customized pipeline to obtain gene expression matrices (Zheng et al., 2017). After the raw sequencing data were generated on the Illumina platform, they were converted to FASTQ format. Quality control was performed using the fastp tool to remove low-quality reads, adapter sequences, and poly-A tails. Cleaned reads were further processed using SeekSoul® Tools for quality control and aligned to the reference genome GRCm38 (mm10) using the STAR software. Cell barcodes and unique molecular identifiers (UMIs) were identified, and an initial gene expression matrix was generated.

### 4.9 Data processing and quality control

Data dimensionality reduction and clustering analysis were performed using the Seurat R package (Stuart et al., 2019). First, the UMI count matrix was imported into the R environment, and a Seurat object was created. During the quality control step, cells with fewer than 30,000 UMIs and gene counts between 200 and 5,000 were retained, while cells with mitochondrial content greater than 10% were excluded. A minimum UMI count of 300 was applied to filter out low-quality cells, removing any cells with nCount_RNA ≤300. The remaining cells were normalized and scaled using the NormalizeData and ScaleData functions. To mitigate batch effects, the Harmony algorithm was applied to integrate data from the GAL and WT groups. Subsequently, the top 2,000 highly variable genes were selected for principal component analysis (PCA) using the FindVariableFeatures function. Nearest-neighbor relationships were determined using the FindNeighbors function, and cell clustering was performed with the FindClusters function, setting the clustering resolution to 0.5. The final clustering results were visualized in a two-dimensional space using the UMAP algorithm.

### 4.10 Differential gene expression analysis and cell type annotation

Differential gene expression analysis was conducted using the FindAllMarkers function of the Seurat package, with the Wilcoxon rank-sum test. The top five most significantly differentially expressed genes (based on log2 fold-change) for each cluster were selected and annotated using the CellMakerDB and PanglaoDB reference databases. Each cell cluster was annotated to a specific cell type (e.g., Kupffer cells, hepatocytes) based on characteristic marker genes (Andrews et al., 2022; MacParland et al., 2018). Gene expression patterns were visualized using the FeaturePlot and VlnPlot functions. Volcano plots illustrating the expression trends and significance of differential genes were generated using the ggplot2 R package.

To exclude batch- or sample-driven effects, we examined sample-colored UMAP plots and confirmed that hepatocytes from all four mice contributed to each of the four subtypes. No subtype was enriched in a specific sample, indicating that the Hep1–Hep4 structure was not driven by sample-specific or batch-related variation.

### 4.11 Functional enrichment and pathway analysis

Gene Ontology (GO) enrichment analysis was performed on the differentially expressed genes using the clusterProfiler R package (v3.12.0) (Wu et al., 2021; Yu et al., 2012). Differentially expressed genes from each cell type were extracted and filtered using the FindMarkers function. After adjusting for multiple testing with the Benjamini–Hochberg method, GO terms were evaluated for significance, and the results were visualized with dot plots to highlight biological pathways significantly enriched in different cell types.

### 4.12 Intercellular communication analysis

To explore intercellular communication between GAL and WT groups, ligand-receptor interaction analysis was conducted using the CellChatDB database (Jin et al., 2021; Troulé et al., 2025). Ligand-receptor pair interaction scores were calculated by multiplying the expression percentage of each ligand-receptor pair. A significance threshold of p < 0.05 was applied to determine the most relevant interactions. Detailed information on ligand-receptor pairs is provided in the Supplementary Material.

### 4.13 Detection of liver index, serum ALT and AST levels

To evaluate liver function, we selected WT and GAL mice of similar age (five mice per group) and performed blood collection via orbital venous puncture following anesthesia. Serum was extracted by centrifugation at 3,000 rpm for 10 min at 4 °C and then stored in fresh EP tubes. Subsequently, the serum samples were analyzed using a veterinary biochemical analyzer (URIT, CA-180Vet).

Post blood collection, the mice were humanely sacrificed, and their livers were carefully excised. The livers were rinsed with 0.9% saline to eliminate residual blood and gently blotted dry. The liver weight was precisely measured to compute the liver index. Liver Index (mg/g) = Liver Weight (mg)​/Body Weight(g).

### 4.14 Immunofluorescence and Western blot analysis of HGF and VEGF pathways

To validate the scRNA-seq results, immunofluorescence (IF) staining and Western blot (WB) experiments were performed to evaluate the HGF and VEGF signaling pathways.

For IF staining, liver tissues were fixed in 4% paraformaldehyde, embedded in paraffin, and sectioned at 4 μm. After deparaffinization and antigen retrieval (sodium citrate buffer, pH 6.0, 95 °C, 15 min), sections were blocked with 5% BSA (45 min) and incubated overnight (4 °C) with primary antibodies (HGF, VEGFA, and KDR (Servicebio), and c-MET (BOSTER, Clone#BFA-13),1:200). Fluorescent secondary antibodies (1:500) were applied for 1 h at room temperature, followed by DAPI counterstaining (15 min). Slides were mounted with anti-fade medium and imaged using a confocal microscope.

### 4.15 Statistical analysis

All data analyses in this study were performed using GraphPad Prism nine software. Comparisons between two groups were conducted using Student’s t-test. Data are presented as mean ± standard deviation (X ± SD). Statistical significance was considered when p < 0.05. For single-cell analysis, appropriate statistical tests were applied based on the nature of the data, as detailed in the relevant sections of the manuscript.

## Data Availability

The data presented in the study are deposited in the GEO repository, accession number GSE313046.
