# Supplementary material for: Single-cell transcriptomics reveals mechanisms of Galt gene editing–induced liver injury involving HGF–VEGF–mediated intercellular signaling in mice
Source: Front Cell Dev Biol. 2026 Jan 15;13:1729321. doi: 10.3389/fcell.2025.1729321 (PMC12851954; doi:10.3389/fcell.2025.1729321)
Supplement: Supplementary file 1 [file Table1.docx]

Supplementary Table 1. Primer sequences for genotyping of *Galt* mouse

| Name | Sequences (5'-3') |
| --- | --- |
| Galt-M-ES-1 | CTGAGCGTGATGGTGAGTC |
| Galt-M-EA-1 | GGTAGTAGTGGGCGTGGA |
