# Supplementary material for: Single-cell transcriptomics reveals mechanisms of Galt gene editing–induced liver injury involving HGF–VEGF–mediated intercellular signaling in mice
Source: Front Cell Dev Biol. 2026 Jan 15;13:1729321. doi: 10.3389/fcell.2025.1729321 (PMC12851954; doi:10.3389/fcell.2025.1729321)
Supplement: Supplementary file 2 [file Table2.docx]

Supplementary Table 2. QPCR primer sequences for *Galt* mouse

| Name | Sequences (5'-3') |
| --- | --- |
| Galt-1-F | GCTCCTCAGGAAGGAACGTC |
| Galt-1-R | CAGCCCATGGAGTAGGGAAAG |
