# Supplementary figures and images for: Single-cell transcriptomics reveals mechanisms of Galt gene editing–induced liver injury involving HGF–VEGF–mediated intercellular signaling in mice
Source: Front Cell Dev Biol. 2026 Jan 15;13:1729321. doi: 10.3389/fcell.2025.1729321 (PMC12851954; doi:10.3389/fcell.2025.1729321)

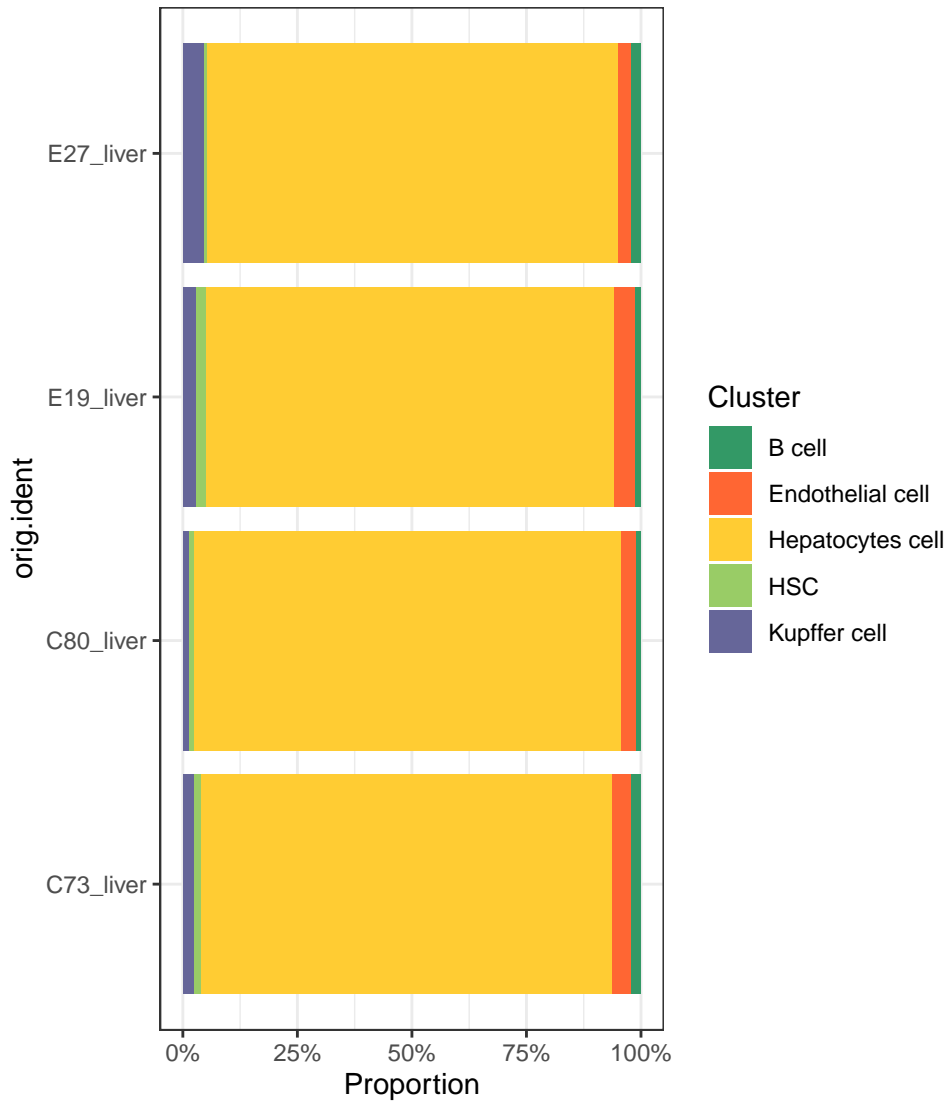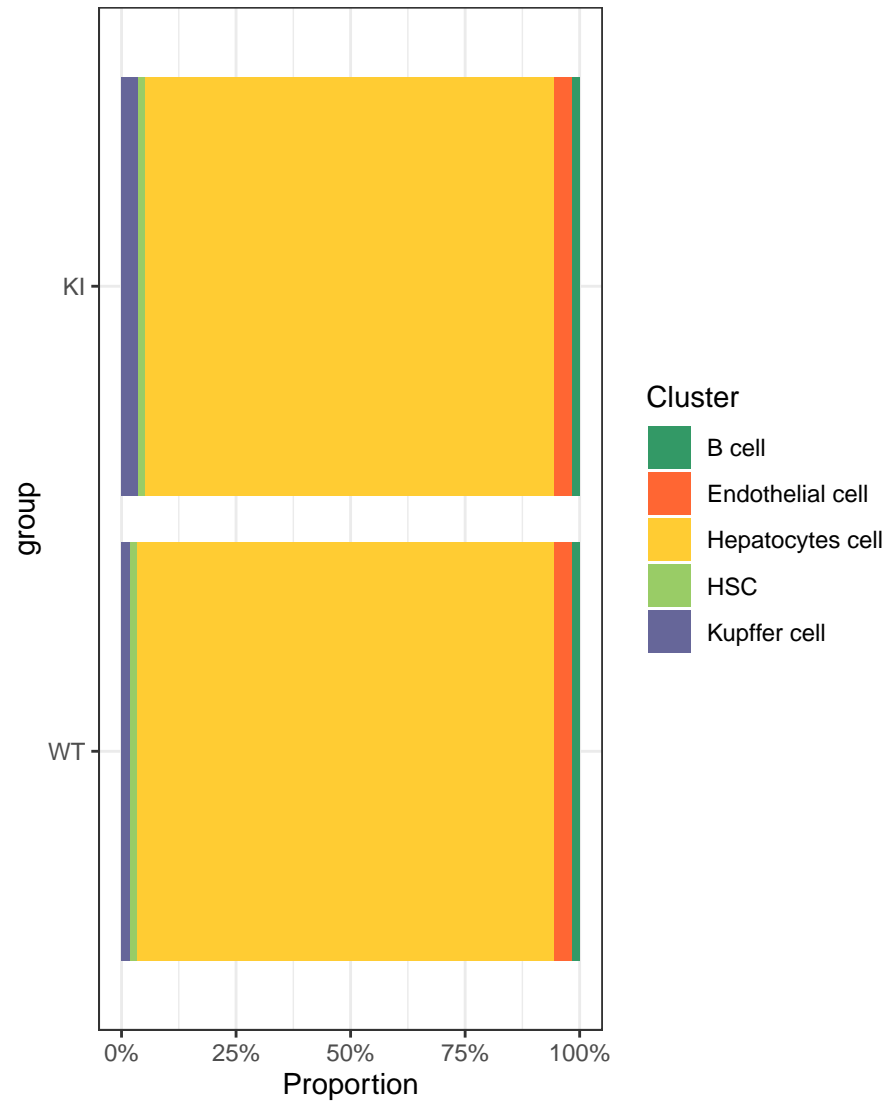

Supplement: Supplementary file 3 [file DataSheet1.pdf]
